# Conceptual, Regulatory and Strategic Imperatives in the Early Days of EEG-Based Biomarker Validation for Neurodevelopmental Disabilities

**DOI:** 10.3389/fnint.2019.00045

**Published:** 2019-08-21

**Authors:** Joshua B. Ewen, John A. Sweeney, William Z. Potter

**Affiliations:** ^1^Department of Neurology and Developmental Medicine, Kennedy Krieger Institute, Baltimore, MD, United States; ^2^Department of Neurology, Johns Hopkins University School of Medicine, Baltimore, MD, United States; ^3^Department of Psychological and Brain Sciences, Johns Hopkins University, Baltimore, MD, United States; ^4^Department of Psychiatry, University of Cincinnati, Cincinnati, OH, United States; ^5^National Institute of Mental Health, National Institutes of Health, Bethesda, MD, United States

**Keywords:** biomarker, EEG, validation, autism, neuropsychiatry

## Abstract

Biological treatment development for syndromal neuropsychiatric conditions such as autism has seen slow progress for decades. Speeding drug discovery may result from the judicious development and application of biomarker measures of brain function to select patients for clinical trials, to confirm target engagement and to optimize drug dose. For neurodevelopmental disorders, electrophysiology (EEG) offers considerable promise because of its ability to monitor brain activity with high temporal resolution and its more ready application for pediatric populations relative to MRI. Here, we discuss conceptual/definitional issues related to biomarker development, discuss practical implementation issues, and suggest preliminary guidelines for validating EEG approaches as biomarkers with a context of use in neurodevelopmental disorder drug development.

## Introduction

Pressing needs in clinical care and concerns about low-yield clinical trials in neurodevelopmental disabilities (NDD) have increased enthusiasm for brain-based biomarkers, with particular additional challenges in neurodevelopmental disorders (NDD) (Sahin et al., 2018). The current push for biomarker development in the realm of brain disorders follows on the heels of several well-known failures in clinical trials in genetically more homogeneous disorders (Berry-Kravis et al., 2012; Hagerman et al., 2018). While there is high enthusiasm for biomarkers for NDDs, the field is just at the beginning of establishing utility of biomarkers for guiding selection of therapies and the patients most likely to benefit in clinical trials. Therefore, the aims of this paper are to help guide early-phase efforts in this area by providing a conceptual framework for planning biomarker validation research, suggestions for early phase investigation strategy and an early framework of thresholds for determining successful reliability/validation, and to explore issues specific to the use of EEG.

These trial failures have been difficult to interpret and seem to have lower-than-expected statistical power due to unpredictable responses to interventions (including placebo), which are in turn due to patient heterogeneity that has not yet been differentiated. The last few decades have made clear that there is poor correspondence across levels of analyses in behaviorally defined NDD (“degeneracy”): patients with a particular genotype may have a range of behavioral phenotypes as in Fragile X syndrome (FXS), and a single behaviorally defined diagnosis [e.g., autism spectrum disorder (ASD)] may be caused by a range of genetic alterations (e.g., Angelman, tuberous sclerosis, FXS, multiple risk loci). Situated between genotype and clinical phenotype are molecular pathways and cellular processes most relevant to the mechanism of action of pharmacological interventions—so called *intermediate phenotypes.* In behaviorally defined disorders and behavioral/cognitive therapies, the most relevant level of intermediate phenotype may be at the cognitive level (Morton, 2005). Because interventions assessed in clinical trials may be effective (or, alternatively, dangerous) for a latent sub-group within a behavioral or genetic diagnosis, there is substantial risk that a true benefit for such a sub-group could be statistically overshadowed by a null effect in most subjects (Type II error). The hope placed in biomarkers is that they can better report on the level of this treatment-linked intermediate phenotype, and thus refine inclusion/exclusion criteria or generate a stratification approach. A solid example comes from epilepsy: the clinical (behavioral) description of a seizure can be misleading in terms of choice of treatment. A generalized tonic-clonic seizure (GTC) may result either from seizure activity arising in the brain all at once, or seizure activity arising from one spot in the brain and spreading quickly across the brain; different medications are effective for each type. These two mechanism are better separated by EEG results than by clinical (behavioral) descriptions of the seizure. The EEG findings therefore, represent a treatment mechanism-relevant intermediate phenotype useful for guiding optimal clinical therapy and testing of novel agents.

Consider clinical trials in FXS, for which a seemingly promising treatment was brought forward unsuccessfully based on compelling data from a genetic animal model (Berry-Kravis et al., 2012) to appreciate how the absence of a biomarker limits the interpretation of results. In the FMR1 knock-out (KO) mouse model of FXS, metabotropic glutamate receptor type 5 (mGluR5) antagonists produce beneficial neurobiological and behavioral effects. No translational biomarker of functional brain data, such as EEG, were collected in the mice, or in the decisive clinical trial. Without a translational biomarker establishing that some desired brain effect was achieved or a clinical biomarker for stratifying individuals based on pre-treatment functional brain alterations related to mGluR5 alterations, it was impossible to conclude whether mGluR5 antagonism did anything to brain function and relate any such effect to clinical outcome. If EEG biomarker data from both the KO mice and the enrolled patients had been available, the extent to which they would have shown similar EEG alterations would have been informative (Ethridge et al., 2017; Wang et al., 2017; Lovelace et al., 2018). While is not yet clear if a pattern of EEG biomarkers reflects a mediator of the effect between mGluR5 antagonism and behavioral change, variable treatment response might be accounted for by those with a better clinical response in those who had abnormal biomarker values before treatment. This pattern may suggest a pathway for using an EEG biomarker for patient stratification or inclusion in future trials.

Further, if in humans, we were confident that the mGluR5 antagonist modified the biomarker in the same way that it did within the mouse model, industry leaders might be more willing to invest in finding out whether a meaningful clinical benefit requires longer treatment, inclusion of a specifiable subgroup of patients with the target syndrome and/or adjunctive behavioral treatment in future trials. Alternatively, no or very limited change in the EEG-based biomarker would argue against pursuing mGluR5 antagonism as a means of altering brain function, especially if there were additional data establishing that the full range of receptor occupancy of the antagonist had been explored using an appropriate positron emission tomography (PET) ligand.

These possibilities illustrate the multiple ways in which biomarkers might facilitate drug discovery programs. At a more rigorous standard, biomarkers validated as surrogate endpoints could reduce expense by identifying in early phase 2 studies the drugs unlikely to translate from mouse to human in a clinically effective manner. Imagine an EEG method that predicts or reports, with high sensitivity and specificity, successful modulation of the mGluR5 system within individuals with FXS. A conventional clinical trial using a single agent might require months of intervention before the robust behavioral or cognitive effects of the drug could be achieved. By contrast, a *sensitive*, validated marker of mGluR5 modulation could allow fast-fail testing of multiple drug candidates, eliminating those which fail to modulate mGluR5 in humans. The presence of target engagement, however, would not necessarily ensure that the compound could safely create the clinical outcomes of interest, but at least large-scale clinical trials could be focused on a biomarker determined dose range for testing drug efficiency.

While genetically homogenous disorders, such as FXS, have a “head start” in identifying mechanisms for targeted drug therapies, biomarkers can still be relevant for identifying processes to target given that behaviorally defined disorders given their often have diverse behavioral presentations indicating that factors beyond a specific genotype are at play. A theory of ASD which has been gaining traction over the last decade specifies that the behavioral phenotype results from an imbalance in inhibitory and excitatory (I/E) processes toward increased neural excitability (Rubenstein and Merzenich, 2003; Belmonte et al., 2004; Ajram et al., 2019). Belmonte et al. (2004) linked these findings with a cognitive model suggesting that decreased inhibition results in dysfunction of early attentional mechanisms and subsequent “overload” of later, capacity-limited processes. They proposed that such physiological-cognitive changes would result in a greater amplitude of event-related potentials (ERPs) captured during relevant tasks. If this could be established, one might select agents which facilitate inhibitory pathways using normalization of ERP amplitude as a read-out.

To date, successful development and validation of biomarkers has usually depend on demonstration of a relationship to some tissue pathology [e.g., plaques and tangles in Alzheimer’s disease (AD)] or a robust functional measure that can be related to some pathological event (e.g., blood pressure and stroke). In the case of AD, before current biomarkers were developed, a definitive diagnosis depended on findings at autopsy. AD biomarkers, such as PET imaging and cerebro-spinal fluid (CSF) assays, are now established as sufficiently predictive of autopsy findings to serve as entry criteria in many clinical trials as well as providing a basis for new diagnostic criteria that included biomarkers (Jack et al., 2018; Veitch et al., 2019).

Advances in the mechanistic knowledge of NDD, even in the absence of known brain tissue pathologies, coupled with advances in EEG analysis technology, have raised the hope of identifying EEG-based biomarkers to increase the yield of clinical trials and ultimately enhance clinical care. The question arises as to whether EEG represents a good investment as a potential biomarker. This question is poorly posed, as EEG is a technology rather than a specific biomarker. An infinite number of parameters can be derived from the EEG signal in both task-locked and spontaneous recordings: time-domain evoked- and ERPs, spectral power, entropy, cross-frequency coupling, and a wide range of different connectivity metrics (Cohen, 2014). Each approach needs to be validated in each individual context (e.g., patient group, treatment) and will rise or fall in that context on its own merits. However, to answer the question as to whether EEG as a technology holds promise as a basis for biomarkers, one need only consider that EEG has been *the* technology *par excellence* (apart from the neurological exam and psychometric testing) for measuring CNS physiology in the clinical setting for the better part of a century. Not only clinical EEG, but somatosensory evoked potentials, motor evoked potentials, brainstem auditory evoked potentials and visual evoked potentials have had unparalleled tenure meeting the high bar of clinical validation.

The question at the current time is whether new EEG approaches will have the reproducibility (reliability) and discriminatory ability (validity) to serve a useful purpose in drug trials for NDD. In the best work to date, biomarker development and validation has taken cues from decades of experience with clinical test validation in the fields of psychometrics and clinical laboratory medicine (Lord, 1955). However, at the current stage of research progress, specific issues related to validation in the context of NDD and EEG are beginning to be grappled with. The vision is that biomarkers can fill a need for reducing uncertainty in clinical care and clinical trials. Poorly validated biomarkers, however, can be expensive and time-consuming wastes of subject and investigator time. To achieve success in biomarker development, it is important that biomarker validation proceed systematically and rigorously to establish utility/validity in the context of performing a specific function in order to be accepted by clinicians, the pharmaceutical industry, and the FDA and related agencies world-wide. With this background in mind, the primary goal of this manuscript is to identify conceptual, strategic and regulatory issues relevant to beginning the path toward valid biomarkers for behaviorally defined NDD and to propose solutions to the many obstacles to success.

Many things are regularly said about what we hope biomarkers will be able to do: that they will offer a highly specific index of one particular molecular or cognitive mechanism, that they can reframe our nosology in a more productive way, or that they will be able to transcend “squishy” outcome measures. We return to these commonly held beliefs over the course of the paper, but begin by offering a concrete definition of “biomarker” and “validation,” in line with how clinical laboratory tests have long been validated. A biomarker is simply *a read-out that empirically provides an estimate of a reference test* (i.e., a previously established diagnostic determination or treatment outcome), under specific operating conditions (specific patient criteria, including age group, symptomatology and/or diagnoses; a specific intervention, where relevant; a specific function, such as prediction of response; and a specific machine and analysis pipeline). Validation establishes how good the estimate is (i.e., sensitivity and specificity. The requirements and logic of a validation study, specifically targeting EEG-based biomarkers, is covered elsewhere (Ewen and Beniczky, 2018), and necessary components *for diagnostic biomarkers* (but not other types of biomarkers relevant to clinical trials) are defined by the STARD checklist (Bossuyt et al., 2015).

A process similar to validation is *qualification*, which refers to a regulatory processes within FDA for judging the effectiveness of a set of biomarkers; it explicitly differs from validation in that qualified (and not only-validated) biomarkers need to be shown to function independently of the technology and precise procedures used (Califf, 2018; CDER Biomarker Qualification Program, 2019). The goal of the qualification process is to allow biomarkers to be used in clinical trials without regulatory endorsement of similar biomarkers individually within each new clinical trial. Validation and qualification differ in that validation occurs in the scientific literature using appropriate psychometric procedures, whereas qualification is currently achieved via consensus panels. For context, only 8 biomarker families have been qualified by FDA, and none for brain-based processes. In the case of AD, amyloid measures have not yet been qualified, despite being in widespread use.

The field has not yet progressed to the point where EEG-based biomarkers in NDD are being routinely validated, and it is informative to consider the “pre-validation” types of studies that are occurring currently (Table 1). We may call these studies *biomarker discovery.* Discovery includes two-groups comparisons of some physiological measure as a dependent variable, or the demonstration that a certain EEG measure correlates with a clinical measure within a patient group. Such studies do not inherently meet the rigorous requirements of validation studies for three reasons: (1) the demonstration of *group* differences in a dependent variable is a lower statistical bar than showing accurate classification at the *individual* level, (2) two-group studies often by design refine the clinical and especially the control samples, whereas validation studies face the more daunting task of classification using groups that encompass all of the real-world patient heterogeneity that will be faced by the clinical trialist or the clinician, and (3) validation studies require setting a threshold based on a “training sample” and replication in the form of a “test sample” (Ewen and Beniczky, 2018).

**TABLE 1:** Types of Studies Related to Biomarker Development.

**Study Type**	**Form**	**Implication**
Biomarker Discovery	Two-group comparisons Correlation between physiological (EEG) measure and clinical variable Data-driven cluster identification Identification of EEG measure in which clinical group is in tail of normative distribution	*A priori* information to suggest that a physiological metric may be a promising candidate for validation
Biomarker Reliability	Test-retest measurement of biomarker read-out	Studies demonstrating poor reliability are adequate to exclude a biomarker candidate from further consideration.
Biomarker Validation	Data collection in training sample. Determination of optimal threshold. In test sample, calculation of sensitivity/specificity.	Adequately validated, a biomarker is ready for use *within the constrained context under which it was validated.*
FDA Biomarker Qualification	Similar to biomarker validation, but not limited to a single methodology or analysis pipeline	Allows the biomarker to be used in FDA studies without re-validation.
Establishing Biomarker as Tool for Measuring an Underlying Physiologic Process in Multiple Contexts	Not a study *per se*, but an accumulation of mechanistic and validation results for a single physiological metric under different contexts of use (COU), different disorders, different age groups and different therapeutic agents	Cross-linked knowledge that will allow us to propose, with some confidence, utility of the biomarker in an even greater range of applications

Another class of biomarker discovery study is data-driven identification of clusters within a particular physiological read-out (i.e., at the intermediate level of the biomarker). While such studies inherently work at the individual level and the overarching sample often contains a great deal of heterogeneity, these clusters are not of value until it has been shown that they are (1) replicable and (2) represent a clinically meaningful heterogeneity (e.g., predicting a response to a particular therapy). The empirical demonstration that data-driven clusters predict some clinically meaningful outcome is the work of validation, whereas the identification of the clusters in the first place is discovery. Efforts are underway and show promising results in neighboring fields. For example, data-driven “biotype” clusters have been identified in EEG and cognitive data in individuals with psychosis syndromes (Clementz et al., 2016). The groups were primarily differentiated using EEG data, with one group showing increased responsiveness to sensory input and increased intrinsic neurophysiological activity relative to healthy controls, and a second group showing reduced responsivity sensory input, reduced intrinsic activity and reduced cortical volumes relative to healthy controls. Inferential statistical tests, such as meaningful heterogeneity and mixture models (Anderberg, 1973; Pauler et al., 1996; Sun et al., 2018), can help establish whether these clusters are likely by chance. This step opens the door to determining whether membership in a cluster better predicts natural history and treatment responsiveness in individuals with psychosis than does clinical diagnostic categorization. In this instance, even if the EEG measure overlaps with normal functioning in some cases, and approximately 1/3 of patients do not show either pattern or difference from healthy controls on these measures, having high or low values might be predictive of response to one or another class of medications.

A related type of discovery study is one in which it is shown that some clinical group occupies the tail of a distribution of a normative sample, on some EEG metric. As with cluster analysis, these data would serve as preliminary evidence that the biomarker may index something of relevance to the clinical group, but it does not specify what the utility of this information may be. All of these biomarker discovery approaches generate potentially important motivation for biomarker validation, but they are insufficient in and of themselves.

Between biomarker discovery and biomarker validation lie *reliability studies*, which demonstrate that a particular biomarker (within a particular context) is reproducible (test-retest reliability) and insensitive to factors which we hope would not affect it, such as site or specific technologist (inter-rater reliability). Reliability studies, unlike validation studies, do not require demonstration that the biomarker candidate estimates the reference test (e.g., clinical outcome). While reliability is insufficient for validation, it is, however, *necessary:* reliability sets the mathematical ceiling for validity. Therefore a biomarker candidate can be efficiently excluded prior to a full validation study based solely on poor reliability. In a reliability study, two measurements can be take in the same day, whereas a validation study could take years to show that a biomarker measurement at the outset predicts an outcome years later. We argue it is this is the stage of development—assessment of reliability—where the field should currently have a focus of attention, both in terms of rapidly screening biomarker candidates as well as for establishing field-wide, empirically derived guidance. Some tentative proposals for steps in this direction are made in the final section of this paper.

On the other hand, going even beyond the level of knowledge required by validation studies, we also begin to imagine what it would take to develop biomarkers that are so well validated, in so many clinical groups and contexts that we can begin to understand them as a representation of a pathological mechanism, like cholesterol in heart disease or a blood sugar in diabetes mellitus. Such an outcome would require cross-linked knowledge and iterative studies (Woo et al., 2017)—both mechanistic and validation—that would transcend the “single-use” biomarker validation studies that form the core of the current discussion.

One crucial aspect this shift from discovery science to biomarker development efforts is to consider data at the individual participant rather than as group means. Biomarkers need to be applied to individuals, and their utility for some context of use needs to be established with such data to demonstrate prediction of outcome, dose optimization, etc. Data in discovery studies are rarely reported from individuals, and this limits existing literature in establishing promising parameters for consideration as targets for biomarker development in future studies. Individual data are also important for identifying cut points for decisions (increase dose, stratify for trials) and to examine distributions for a group of outliers from the range of healthy controls, or bimodality/discrete heterogeneity that would suggest subgroups that could be examined separately.

The goal of this manuscript is not to review the state-of-the-art in EEG-based biomarkers in NDD; several such reviews are in the recent literature (Wang et al., 2013; Takarae et al., 2016; Gurau et al., 2017). Our goal, rather, is twofold: in the first half, we dissect commonly held conceptual issues specific to NDD-focused and EEG-based biomarkers. Specifically, we discuss knowledge requirements for reliability and validation studies. We consider factors of heterogeneity/comorbidity, development and state/task performance.

In the second half of this manuscript, we argue for a strategic approach that includes academic, industry and governmental stakeholders (including NIH and FDA). We talk about the many significant advantages of EEG for biomarker development in NDD populations. We offer tentative reliability thresholds a promising biomarker should meet for it to be intensively studied and used for biomarker purposes. While the suggested criteria are preliminary and will evolve over time, we believe that they represent a good starting point, based on experience across fields, and highlight the need for operational standards. We argue that much research to date aiming to advance biomarkers of brain function has suffered from a lack of alignment on performance characteristics (“psychometrics”) of the methods, the rare use of biomarkers in randomized clinical trials, especially in pediatric neuropsychiatry, and emphasis of grant funding on using technologies at hand to explore disease mechanisms. These latter studies often occur in the context of small single-site studies using “pure” samples with restricted recruitment criteria and novel neuroscience techniques. These studies are rarely followed by larger multi-site studies that take into account the heterogeneity and confounds encountered in typical clinical populations in order to validate measures as biomarkers for broad use. Large multisite studies can accelerate testing of a range of measures to select which provide information that is truly useful for a context of use, and to identify biologically distinct subgroups in behaviorally defined conditions. Such first steps are needed for biomarker discovery, but are insufficient toward validating biomarkers to improve clinical trials, enhance the replicability of studies of disease mechanisms and ultimately inform clinical practice for the general population. For years, the FDA has pointed out (Mullard, 2019) that consortia models are far more likely to succeed in developing data in support of biomarkers.

### Criteria for Validation

**FIGURE 1:**
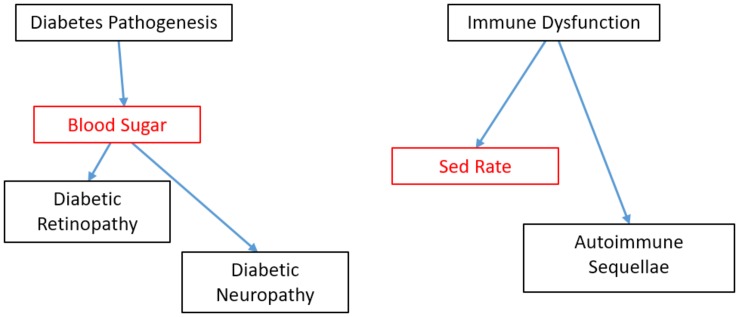
Causal models of blood sugar used as a biomarker In diabetes and sed rate as a biomarker in autoimmune disorders. Biomarkers shown in red. Mechanistic knowledge is neither necessary nor sufficient for demonstrating validity of a biomarker candidate within a particular COU. However, because we know that blood sugar has a direct causal role on certain complications of diabetes, this knowledge opens up the reasonable possibility that blood sugar could be successfully validated as a surrogate biomarker as well as a diagnostic, pharmacodynamics/response and monitoring biomarker. By contrast, little It known about the relationship between sed rate, most used as a monitoring biomarker, and the causal path of clinical sequellae in autoimmune disorders. This absence of information does prohibit the sed rate from being validated as a diagnostic, response or monitoring biomarker; it simply means there is less *a priori* knowledge going into those validation studies.

As discussed above, the core of validation is the empirical demonstration that a biomarker performs its specified function at some criterion level. As such, only an empirical statistical relationship between biomarker and reference test is needed; no mechanistic knowledge is required. Indeed, some of the most widely ordered and demonstrably useful tests in the history of modern medicine are not supported by an understanding of how the test read-out relates to the pathophysiology of the disorder. The Westergren erythrocyte sedimentation rate (ESR or “sed rate”) has been a widely utilized test in the care of patients with possible and actual inflammatory conditions, yet the rate at which red blood cells settle in test tubes is only indirectly related to the pathogenesis of those inflammatory conditions (Figure 1). We return to causal diagrams later, when envisioning sets of biomarkers that begin to transcend single applications.

While the process of EEG-based biomarker validation has been explicated elsewhere (Ewen and Beniczky, 2018), it may help to organize our thinking by considering four “ingredients”: a specified EEG measure derived from the raw data; a context of use (COU), a reference test for comparison, and selection of population.

#### What to Measure

The EEG is a complex signal, and there is no end to the mathematical techniques that can be applied to it (Cohen, 2014). This poses the challenge of identifying which specific EEG measures and what specific behavioral paradigms from this infinite list stand the greatest chance of successfully proving valid for their intended purpose. Biomarker discovery studies, of the type laid out in the Introduction, identify potential biomarker candidates. Scientific studies of the pathogenesis of the disorder in question or the pharmacological mechanism of the proposed treatment also allow one to identify candidate biomarkers that seem most promising. Because EEG-based techniques are widely used as scientific tools in the study of NDD mechanisms both at the behavioral/cognitive and molecular/circuit levels, there is an active literature for first-stage evaluation of the most disorder-relevant EEG measurements and paradigms that are biomarker candidates.

There may be a difference in how this step is approached in behaviorally defined disorders *vs.* genetically defined disorders, because of the “convergence point” of both the pathophysiology of the disorder as well as of therapies. Genetically defined disorders typically implicate a molecular pathway in their pathogenesis, and current-day efforts at treatment are often pharmacological, targeting the pathogenic pathway. The EEG biomarkers most tapped in an effort to specifically index these pathways tends to be low-level sensory responses, for which animal models provide extensive information about relevant neurophysiology and neurochemistry. Low-level sensory responses are biologically “closer” to molecular, cellular and circuit processes, and can also be performed on relevant animal models, facilitating direct translational application of testing and measurement procedures. A specific example and what is needed to advance it for some COU is provided later.

Behaviorally defined disorders, such as ASD, likely have multiple potential genetic, molecular and circuit deficits that all result in a more or less common cognitive deficit [though see also (Waterhouse et al., 2016)]. Because we cannot currently parse or subdivide the potential “lower level” causes, we are currently left with conceptualizing and managing the disorder on a cognitive level. The differential diagnosis (e.g., social-pragmatic language disorder, intellectual disability) is also defined at the cognitive level. The partially effective therapies for core symptoms to date are behavioral in nature, such as Applied Behavior Analysis (Lovaas, 1987). As a result, it seems reasonable that one would have the highest probability of validation success for a biomarker candidate that was designing on a cognitive intermediate phenotype, taking into account known and theorized factors about the specific disorder and intervention. The ERP paradigm involving looking at faces discussed later is an example involving observable and theorized aspects of ASD.

Task-related EEG measures have been a mainstay of experimental (cognitive) psychology and psychophysics for decades (Luck, 2014), parsing such models in both “health” and disorder. Therefore, we can leverage existing scientific tools from that literature for consideration as biomarker candidates. We are hesitant about the use of “out-of-the-box” paradigms to elicit certain ERP components *vs.* developing or selecting tools based on intended use and clinical considerations. For example, the P3 component (a/k/a P300) is elicited in oddball paradigms. Tasks can be designed, however, to elicit the P3 to specifically index stimulus discrimination effects (Patel and Azzam, 2005), expectancy effects (Wu and Zhou, 2009), contextual effects (Polich, 2007), memory recall effects (Fabiani et al., 1986), resource allocation effects (Kida et al., 2004), and processing efficiency effects (van Dinteren et al., 2014). A biomarker is more likely to be shown to be empirically valid if a task is designed that takes into account data and theory regarding the disorder under study, the cognitive endophenotype under study, as well as intended or known effects of the study therapy.

Additional EEG metrics and their corresponding constructs in neuroscience, such as cerebral connectivity (Vasa et al., 2016; O’Reilly et al., 2017), are currently under scientific investigation and could potentially serve as biomarker candidates in the future. This line of work is now widely used in the fMRI and EEG literature, but its use for biomarker purposes is largely unexplored.

Recording standards and procedures, and the analysis pipeline are also specified within this element. Multi-site studies such as the (ABC-CT) are taking the lead in developing rigorous standards. This effort follows in the footsteps of long-standing standards in clinical EEG (Sinha et al., 2016) and more recent guidelines in EEG-based research (Picton et al., 2000; Webb et al., 2015).

#### Context of Use

The second ingredient for validation is the COU. COU is FDA language for the specific *function* that the biomarker performs. FDA and NIH, in their “BEST” (Biomarkers, EndpointS and other Tools) collaboration (CDER Biomarker Qualification Program, 2019) define the multiple types of COU and are critical for preparation for FDA qualification (Table 2).

**TABLE 2:** FDA Biomarker Contexts of Use (COU).

**COU**	**Description**
Diagnostic	Concurrent biomarker that specifies whether or not an individual has a disorder/pathologic process
Monitoring	Concurrent biomarker that concurrently reflects a change in a disease or in a side effect
Safety	Concurrent biomarker that reflects presence/degree of toxicity from an exposure
Response	Prospective biomarker that reflects a response to an intervention; when highly well validated, may serve as a *surrogate endpoint* in a clinical trial
Prognostic	Prospective biomarker that predicts clinical course
Predictive	Prospective biomarker that predicts response to an intervention
Susceptibility/Risk	Prospective biomarker that reflects potential for developing or disease sensitivity to a negative outcome following an exposure

This manuscript focuses on prospective biomarkers in clinical trials. There are some preliminary efforts at diagnostic biomarkers for clinical care in NDD (Loo et al., 2013; Snyder et al., 2015; Ewen, 2016; Gloss et al., 2016). There is at least one prognostic, EEG-based biomarker used in NDD, beyond clinical EEG interpretation: infants born with a port-wine birthmark (PWB) have around a 25% probability of going on to develop the brain involvement that is definitional to Sturge-Weber syndrome (SWS). The use of a quantitative EEG metric, based on a measure validated to measure ischemia during carotid endarterectomy (van Putten et al., 2004), prognosticates which infants are at higher risk and is less expensive and invasive than using MRI (risks associated with sedation and gadolinium contrast administration) and possibly an earlier biomarker, given the rates of MRI false negatives in the first year of life (Hatfield et al., 2007; Ewen et al., 2009).

**FIGURE 2:**
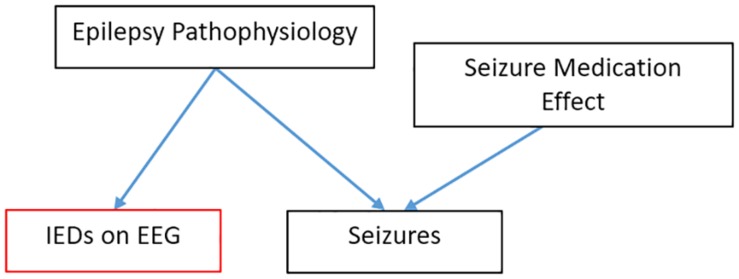
EEG finding can be a good diagnostic biomarker while being a poor monitoring biomarker in epilepsy. In this causal model, lEDs follow from the pathophysiology that causes seizures but are upstream to the effect of seizure medications.

While a particular method or read-out may eventually be shown to function validly in multiple COU, a single validation study reports on performance only within a single COU. Successful performance in one COU does not guarantee adequate performance in another COU (Figure 2). For example, a valid and useful diagnostic biomarker may not be an effective response biomarker. An example from current clinical practice: if a patient is suspected of having epilepsy, we often perform an EEG to look for inter-ictal epileptiform discharges (IEDs)—spikes and sharp waves which indicate an increased likelihood that the spells are epileptic, rather than some non-epileptic “mimic.” IEDs on EEG, while imperfect, are a clinically useful diagnostic biomarker. If our example patient is then diagnosed with epilepsy, the goal of treatment is to reduce or eliminate the seizures. In the process, some seizure medications also normalize the EEG (suppress IEDs), but others effectively reduce seizures without minimizing or eliminating IEDs on EEG. IEDs on EEG, then, are a good *diagnostic biomarker*, but they are a poor *monitoring biomarker* for patients treated with non-spike-suppressing anti-seizure medications.

As the field develops, we can envision biomarkers that have been validated in multiple COUs, and paired with progress in the understanding of how lower-level mechanisms produce the biomarker read-out (Table 1). Imagine an EEG-based biomarker that is similar to blood sugar (Figure 1). Blood sugar has been validated as diagnostic marker for diabetes mellitus. Because we understand how high blood sugar plays a pathogenic, causal role in the complications associated with diabetes, we can we can propose with confidence (and subsequently validate) blood sugar not only as a diagnostic biomarker, but also as a monitoring biomarker. This link would not be true if blood sugar were only a peripheral, epiphenomenological read-out. This mechanistic knowledge can also motivate novel therapeutics (e.g., those which control blood sugar) and subsequently serve as a pharmacodynamics/response biomarker or even surrogate endpoint for this new therapy. However, despite this mechanistic knowledge, sensitivity/specificity need to be calculated separately in each COU (validation). The number of EEG read-outs tightly linked to lower-level mechanisms is small. One auditory ERP paradigm is tentatively becoming linked to a LTP-like mechanism, with systematic studies that showing it is sensitive to the same experimental manipulations as LTP in mouse models (Clapp et al., 2012). If one were to conduct a clinical trial of a drug that is known to affect LTP in animal models and whose mechanism of action to benefit the patient is through modulation of LTP, then using this LTP-sensitive ERP biomarker may give at least some *a priori* confidence that the biomarker will predict or track the efficacy of the therapy.

#### Reference Test

The third element of the validation “equation” is a *de facto* reference test or reference standard, in the terminology of clinical test validation (Bossuyt et al., 2015). Reference standards are often referred to as representing “ground truth” or the “gold standard.” A validation study outputs the sensitivity and specificity with which the biomarker (“index test”) estimates the reference test. The COUs most relevant to clinical trials are the prospective COU: *prognostic* biomarkers (for enrichment in prevention trials), *predictive* biomarkers (for stratifying based on expected sensitivity to treatment), and *risk* biomarkers (for exclusion based on anticipated risk). The value added by the novel biomarker is that it reports earlier than the reference test. The reference standard may therefore be a relatively simple outcome measure, such as a clinical global impression (CGI).

It seems self-evident that sensitivity and specificity can only be calculated relative to some “gold standard.” The reason we make a point of it is in response to an oft repeated hope that a novel biomarker can transcend the limitations of a noisy or subjective reference test, such as the CGI. This hope may be founded in cases where a biomarker is so well validated in multiple contexts, disorders and therapies that it is a proven, faithful representation of a particular mechanism (Table 1; Woo et al., 2017). However, in the case of “single-use” validation studies, it is logically impossible to demonstrate that a novel biomarker is “better” than the reference test against which it is being compared, since it is impossible to disambiguate the uncertainty associated with the novel biomarker from the uncertainty associated with the reference test; this is analogous to being unable to solve a single equation with two unknowns in algebra. Imagine if we had a EEG biomarker which was shown to predict outcome on therapy 12 months before the CGI demonstrated that outcome. There would be some individuals in whom the two tests disagreed. If we took the position that the EEG predictive biomarker were “more correct” than the “squishy” CGI, what data that is “even more true” than the CGI reference test could we even use to demonstrate this was so?

The relationship between biomarker candidates and *concurrent biomarker* reference standards is a bit more complex and will not be fully discussed here. Put briefly, the motivation for developing a new biomarker to substitute for or complement an existing concurrent reference test is because the newer biomarker is less expensive, easier to perform or is less invasive than the biomarker it will replace. Moreover, special issues pertain to reference standards for concurrent COUs specifically in behaviorally defined NDD, and diagnostic biomarkers in particular. However, it is worth mentioning that potential advances in reframing our current diagnostic paradigms to be more in line with evolving therapies could be made via predictive biomarkers. Responsiveness to Intervention (RTI) diagnostic approaches have been used in the context of academic interventions for specific learning disabilities (Ewen and Shapiro, 2008). Because a patient with a NDD may eventually be prescribed both pharmacological and behavioral/cognitive therapies, an RTI framework may be multiaxial and contain multiple, parallel frameworks, one for each of the types of therapy.

#### Selection of Population

The fourth element of the validation “equation” is the selection of the population to be studied (i.e., inclusion/exclusion criteria for the validation study and also patients on whom the biomarker can be validly used in clinical practice or trials). These same inclusion/exclusion criteria and sampling scheme that define the participants/patients for which the biomarker can be validly used in eventual clinical trials or clinical practice. Because we are limiting our discussion primarily to biomarkers which are validated prospectively (prognostic, predictive, and risk COUs), only one group will be recruited; considerations about appropriate comparison groups for validated biomarkers with concurrent reference standards (e.g., diagnostic COU) are not relevant here.

*Heterogeneity* is a potential confound in validation studies that is well recognized in the study of behaviorally defined NDD as well as across neuropsychiatry generally. Aspects of heterogeneity include ranges of severity of core features, presence/absence of non-core but highly penetrant features (e.g., motor dysfunction in ASD) and the presence of comorbidities (e.g., Axis I psychiatric comorbidities). When considering the impact of heterogeneity on biomarker validation, the first point is to restate that a particular biomarker is only valid in implementation for the inclusion/exclusion criteria under which it was validated. While mechanistic science typically seeks “pure” samples to reduce the effect of confounds, biomarkers for advancing drug discovery typically need to seek study participants with more diverse ecological heterogeneity. This heterogeneity then is “baked into” the sensitivity and specificity estimates, which are the end result of validation. Because of this, biomarker studies need to include more messy heterogeneity than projects primarily interested in disease mechanism.

It is possible, however, to improve on gross sensitivity/specificity estimates derived from binary biomarker outcomes by including additional terms in a more complex predictive model. Such terms may and generally should include age, gender, intelligence, duration of symptoms and psychiatric comorbidities in the case of NDDs; the choice of terms will depend on existing knowledge and mechanistic hypotheses about how these factors could influence the biomarker output, but machine learning can readily accommodate such data, given adequate sample size. An interaction term may be critical. Anxiety, for example, may manifest and be due to different mechanisms when co-occurring with ASD *vs.* when occurring in individuals without ASD (Rosen et al., 2018). As a consequence, if one tries to control for a psychiatric comorbidity in a NDD biomarker, it is important to study the biomarker in a 2 × 2 contrast (NDD, psychiatric diagnosis), and to use interaction terms in the predictive model. Similarly, if we hope to account for the effect of medication on the EEG dependent variable, such effects need to be studied both independently and within the context of the disorder of interest.

Certain confounds will require exclusion, such as inadequate visual, auditory or motor function to participate in the biomarker data collection (Picton et al., 2000).

*Development* represents a special case of a confound. We know that many both resting state EEG measures and ERPs vary over the course of development (Tome et al., 2015; Eberhard-Moscicka et al., 2016). While the inclusion/exclusion criteria define the relevant potential patients for biomarker use, *a priori* knowledge about development in neurotypical subjects may itself indicate a need to limit use of a given biomarker only to a relatively narrow age group that does not have significant changes in the EEG dependent variable, or to carefully define developmental progression prior to broad biomarker implementation.

Most biomarker dependent variables represent a measurement at a single point in time. *Monitoring COU* biomarkers, by contrast, measure changes over time. Measurements of such changes will be influenced by test-retest variability, typical developmental changes over long follow-up periods and intervention-related changes. Control measurements over a variety of time courses are necessary to quantify test-retest variability and typical developmental changes for specific COU. There is also, in principle, no reason that diagnostic biomarkers could not be defined by trajectories over time, rather than by point measurements, or that predictive biomarkers showing a small response to a brief treatment challenge could not validly predict a larger response to a longer treatment.

At the current stage of development, most EEG-based biomarker candidates for NDD have only begun to be systematically evaluated for biomarker use. Research efforts typically have focused on a specified age group, in the context of a single disorder, a single therapy (where relevant), and a single specific assay/analysis pipeline – often focused much more on mechanistic science than practical use of measurement for applied biomarker purposes. The latter requires focus on casewise data, utility for prediction or classification, and optimal thresholds for decision making. As we move toward biomarker families that index an important mechanism across multiple conditions, COUs and age groups, we may develop *normative data.* Such studies, as in the field of psychometrics, will require large, heterogeneous groups with random sampling, with sample sizes dependent on the relevant variance (reliability) and effect sizes in the groups.

#### Analytic Approach for Reliability and Validity

Reliability is a precondition for validity. Said another way, it is impossible to detect a meaningful change if the metric varies randomly in the absence of a substantive change of the process that is being measured. Reliability is a metric that is internal to the biomarker itself and does not need to be compared with the reference standard (in fact, the reference standard is taken to be a scalar and not a probability distribution, therefore a reference standard does not have reliability *per se*). Reliability can, however, be compared with that of other, competing biomarkers. Because measurement error and reliable effect size have an inverse relationship, smaller changes can be detected in a measure that has greater reliability. When clinicians or trialists hope that an EEG biomarker will be “less subjective” than, say, parent report, it is increased reliability that they are to a large extent seeking. Because reliability of a certain effect size is necessary for validity at that effect size, it is possible to exclude a biomarker candidate if the reliability is lower than an acceptable threshold. Specific recommendations for EEG-based biomarkers in NDD will be made in the next section.

*Validity:* Many of the clinical electrophysiological tests that have been used in practice for decades have had extremely high sensitivity (like 3 Hz spike-wave for absence epilepsy) or have been definitionally related to their clinical syndrome (Trisomy 21). We cannot assume that current day biomarker candidates, considered in isolation, will have such robustness for syndromal neurodevelopmental disorders, and we need statistical approaches to deal with this reality.

Most analytic approaches for validation have a pipeline of continuous data, binarized data and probabilities. Validation studies require the recruitment of two participant samples with identical inclusion/exclusion criteria: the training set and the test set. Within a training set, continuous data (e.g., ERP amplitude) is collected for subjects in each group; in the case of prospective (prognostic, predictive and risk) biomarkers, group status is assigned retrospectively (good *vs.* bad outcome). receiver operator curves (ROC) allow the break point to be set at a preferred sensitivity/specificity trade-off. The second sample, the test sample, then has the same procedures run, with the same EEG metric, same procedures, same reference standard and same inclusion/exclusion criteria. On this test sample data, the data are binarized via the threshold determined using the training sample, and the true sensitivity and specificity are computed as probabilities. These sensitivities and specificities explicate the uncertainty with which the biomarker estimates the reference standard and serve as the culmination of the validation process.

Criteria for judging minimal data quality standards for a particular set of data from a particular patient/participant to be considered “valid” also need to be explicated within the training sample stage, both from EEG data quality as well as from behavioral performance on any task under which the EEG is recorded.

Biomarkers may be judged not only by their sensitivity and specificity, but by their cost, availability, invasiveness, ease of deployment (including training requirements for staff), rate of data loss to artifact/non-compliance and ability to be tolerated by patients. These considerations can often help decide between two biomarker technologies as most likely to be most efficient for clinical and trial needs. Compared with fMRI, EEG is less sensitive to motion because the electrodes move with the head, and it is far less expensive, therefore more widely available.

#### State-, Performance- and Noise-Related Confounds

**FIGURE 3:**
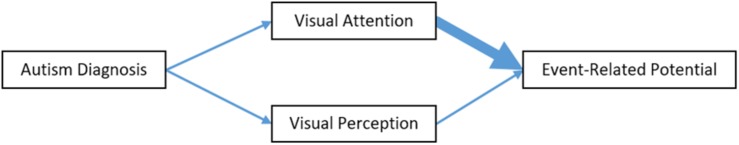
Confounded measure intended to index visual perception. In this example relevant to a diagnostic biomarker, a specific form of Visual Perception alteration is understood to be a consequence of ASD and is intended to be indexed by the Event-Related Potential (ERP). However, Visual Attention (such as looking at the stimulus display) is both necessary for task performance and is also systematically different between the ASD group and the control group. In this example, Visual Attention has a bigger impact on the ERP dependent variably (heavy arrow) than does the Visual Perception ability, and therefore confounds the interpretation of the ERP read-out as a valid measure of Visual Perception.

A variety of confounds commonly encountered in individuals with NDD and in EEG metrics can create problems of variance (reliability) and bias (specificity and subsequently validity). Cognitive electrophysiology biomarkers can be sensitive to processes that are outside the causal chain of (epiphenomenological to) the biological mechanism that is the focus of study—processes which can differ between systematically less- and more-severely affected individuals or treatment responders and non-responders. In the example of Figure 3, the ERP read-out as a valid measure of a particular visual processing mechanism is confounded by a visual attention capacity which is systematically different between groups.

There are at least three approaches to minimizing the effect of artifact and other confounds: utilizing measures insensitive to the artifact generator, using signal processing methods to remove the artifact, and controlling statistically for artifact. The optimal solution is to use electrophysiological metrics which are relatively insensitive to these confounds. For example, the mismatch negativity (MMN) ERP component is minimally sensitive to attention, whereas the P3 component is highly dependent on attention. Auditory perception does not require the orienting of sensory organs in the way that vision does, and therefore auditory tasks may be preferable when testing children who are less able to follow task instructions. EEG and magnetoencephalography (MEG) are silent, making them preferable to fMRI for auditory tasks, and especially for patients with auditory hypersensitivity.

It is critical to study these metrics explicitly in terms of their sensitivity to confounds. A poignant example comes from the fMRI literature, in which it was learned that motion artifact (Power et al., 2012) leads to spurious changes in connectivity measures, which subsequently led to a substantial portion of ASD connectivity literature being called into question (Vasa et al., 2016). Muscle artifact can be a similar issue in EEG studies. When a particular target mechanism or confound is not amenable to direct control, an alternative is to try to equate participant state during individual trials as much as possible. For example, eye tracking can be used to trigger stimulus presentation only when a participant is looking at the screen (Varcin et al., 2016). Behavioral psychological preparation and management during testing can help equate task engagement in a way that is often not directly quantifiable (Paasch et al., 2012). When the EEG biomarker is collected in the context of a psychophysical task, it may be possible to use a staircase method to equate subjects on task performance, to eliminate measured differences that may be due to performance-related mechanisms and not diagnosis-related mechanisms. Parametric studies across a wide range of task difficult are another strategy for dealing with this issue, as it allows brain activity to be modeled across a range of task performance quality.

A final method to control for state/performance confounds is to record behavioral variables during the task and to adjust statistically. In some cases, the behaviors quantified are subjective (e.g., behavioral aide impression of participant engagement); in other cases, they are objective (e.g., reaction times and error rates to the psychophysical task being recorded). Control conditions in psychophysical tasks may help in controlling statistically for confounding processes. There is an added benefit by contrasting conditions within a subject, that any within-subject error term that is common to two conditions is eliminated (Webb et al., 2015).

The number of trials excluded for behavioral (not attended, incorrect task response) and EEG-signal-quality reasons also needs to be tracked, at the very least to make a judgment about which subjects are judged to have inadequate data, invalidating the use of the biomarker for that particular testing session. The same objective criteria for excluding a subject’s data need to be employed both during the biomarker validation study and when the biomarker is eventually used in clinical trials or clinical practice, which involves objective, rules-based definition of acceptable data *a priori.*

Electrophysiology signal quality may differ between groups for reasons that are not clearly known and may not be related to the processes that the biomarker is intended to index (Butler et al., 2017). Additional signal quality metrics are on the horizon. In the meanwhile, it should be pointed out that biomarker studies and mechanistic studies differ in terms of how they are impacted by unaddressed confounds. In mechanistic or treatment studies, where the end result is a binary conclusion (groups do or do not differ in a certain regard), confounds may bias toward a Type I or Type II error. In biomarker studies, the end result is not binary, but statistical measure of uncertainty (sensitivity, specificity), and uncontrolled confounds may simply result in poorer sensitivities and specificities than would otherwise be the cases (assuming random sampling). In some instances, the confounds make the biomarker. The ADHD200 competition was an attempt to discover and validate a fMRI-based diagnostic biomarker for ADHD—and the head-movement variable turned out to be the key predictor (Eloyan et al., 2012)!

Epilepsy, which has increased in recognized prevalence in ASD (Spence and Schneider, 2009; Ewen et al., 2019) and many other NDDs, presents several confounds. First, frank seizures can affect both consciousness/the ability to make volitional responses as well as the EEG tracing. One would suspect that most perceptual/cognitive/motor biomarkers would not be reliable in patients actively having seizures during the recording. The role that IEDs have in alterations of consciousness in the absence of clinical seizures is controversial (Landi et al., 2018). However, patients who have epilepsy but who are not actively seizing also have IEDs in their EEGs (Fisher and Lowenbach, 1934; Gibbs et al., 1935). The extent to which these inter-ictal EEG changes affect (bias) any particular EEG analysis method is an empiric question. Perhaps surprisingly, Key et al. were able to obtain similar ERP waveforms with a similar number of trials from controls and children with Angelman syndrome—a disorder which is known to cause extreme abnormalities of both background oscillatory activity as well as the frequent presence of IEDs, since the IEDs and oscillations are not consistently phase-locked to the stimulus and were therefore canceled out in time-locked averaging. It is probable that spectral (frequency-domain) measurements would be more affected than ERPs in Angelman syndrome. On the other hand, while working on this very manuscript, one of the authors’ (JBE) labs recorded an ERP study in a participant with epilepsy who had IEDs time-locked to and apparently evoked by an auditory stimulus; these focal sharp waves confounded the ERP waveform in certain channels.

In summary, researchers and clinicians desire predictive, prognostic and risk biomarkers to provide an indication of efficacy or side effect earlier than would otherwise be possible, thus making clinical trials more efficient and potentially reframing diagnosis to an intermediate phenotype more tightly related to effective treatments. These biomarkers can also help stratify patients to increase effective power in clinical trials, using the same sample size. While few biomarker candidates are on the horizon for full validation, the simpler assessment of reliability may help cull the heard of candidates. Mechanistic knowledge is not formally required for validation but has the potential to link validated biomarkers to new COUs and can help investigators predict and mitigate certain confounds.

### Developing Paths Forward for EEG-Based Biomarkers in NDDs

As noted earlier, the FDA process, which endorses some specific COU for biomarker qualification, does not have explicitly published requirements. Nor for the broader field is there alignment as to the level of evidence required to judge a biomarker as both sufficiently validated and robust to justify decision making in any interventional study. In order for a biomarker to be used as an inclusion criteria or early intermediate outcome measure, cut off points for decision making need to be specified. And when using a cut-off value in an individual for such uses one wants to have as much confidence as possible that the value truly represents a characteristic of that individual which is potentially relevant to treatment and not due to other sources of variation. In the absence of any EEG based biomarker embraced as likely to currently serve such a role, an initial step in recommending a path forward is to identify gaps in approaches taken to date.

Performance characteristics of single analyte biomarkers in a biofluid such as serum cholesterol are much more straightforward to establish—e.g., standard tube type and processing of sample prior to determination of concentration with clinical laboratory improvement amendments (CLIA) standards in place to provide confidence in reported values—than any functional EEG measure. The wide range of factors that can affect EEG data have been spelled out in the preceding section. Clinical EEG societies specify minimal technical standards (Sinha et al., 2016), and research ERP standards have been published in cognitive psychology broadly (Picton et al., 2000) and for ASD specifically (Webb et al., 2015), but these are not at the level of CLIA standards. It remains to be seen whether they are sufficient for purposes of biomarker qualification and validation or are even followed by most investigators. Given that fMRI-based biomarkers are the other major functional brain measure being pursued in syndromal CNS disorders, we refer the reader to recent reviews of the various roles of fMRI as a functional brain measure applied to drug development (Woo et al., 2017; Carmichael et al., 2018), since these highlight many parallel issues to those arising with EEG. fMRI and EEG though provide very different information. While fMRI provides whole brain coverage and far superior source resolution, the superior temporal resolution of EEG (∼1000Hz *vs.* 1Hz for fMRI) provides a far better characterization of the dynamic interaction of cortical regions and latencies of brain responses, and the biological meaning of frequency information is much clearer than are oscillations in fMRI BOLD signals.

Typical gaps in EEG biomarker development efforts result from a failure to study a sufficiently large and representative slice of the population for which it is ultimately intended. Most preliminary studies of a novel biomarker candidate focus on some small (less than 25 subjects) rarified patient group accessible to a single site and a completely asymptomatic healthy-control group. As a corollary, studies in special populations at sites with staff enthusiastic about and committed to the measure may convey an overly optimistic sense of what percentage of participants can comply with the biomarker procedure and return valid data. This consideration is particularly critical in EEG-based tests for children with neurodevelopmental disabilities (NDD). Task-based EEG measures, particularly those which require behavioral responses in addition to the EEG data collection, set a higher bar for participant compliance than do spontaneous (“resting-state”) metrics.

Differing EEG data-cleaning and processing pipelines are used by different investigators, and it is not clear whether these differences account for differences in reported values and biomarker utility. Variability also occurs because many EEG measures are sensitive to subject state: drowsiness/level of alertness, effortful cooperativeness and degree of relaxation—so how such variables are controlled needs to be clearly defined for reprodicibility. There is also a potential impact of duration of testing, as these factors may become increasingly relevant with longer testing of NDD patients.

At its core, validation requires an evaluation of sensitivity and specificity which are in turn limited by the test-retest reliability for which precise estimates, especially across sites, requires methodologic studies. Ideally, everything relevant to having confidence in reported values should be addressed in the methods section of reports. Fundamental research is needed to investigate explicitly the impact of technical and analytical differences. While equipment manufacturer is assumed to play a far smaller role in EEG output than in fMRI, it would be helpful to know to what extent different EEG amplifiers produce meaningfully different results. Questions also arise about whether activity should be averaged over a prespecified set of electrodes to increase reproducibility, *vs.* selecting electrodes on an individual patient level (through some principled basis) in hopes of increasing SNR. Studies to consider different behavioral test paradigms and different data analytic approaches are thus a crucial part of EEG biomarker development.

In the context of these considerations, performance thresholds or targets for biomarkers likely to be adequate for use in trials and/or qualification by the FDA will need to be refined iteratively with experience. As a starting point, we propose explicit (albeit preliminary) criteria which we hope will drive forward EEG-based biomarker development for drug discovery. To illustrate why we believe that target criteria might be helpful, and to provide and critique examples of biomarker development approaches, we next consider examples from three different classes of EEG based studies—resting-state EEG, ERP to a sensory stimulus anchored in neural systems research, and ERP to a more complex stimulus derived from psychological models and clinical observations.

We start with consideration of resting state EEG studies, using a recent review of relevant published studies in ASD published between 1980–2016 (Gurau et al., 2017). Their summary is instructive with regard to what might be required to nail down an EEG measure as a biomarker at the individual level. All reviewed case-control studies reported some, but not the same, differences between ASD and control subjects. The review considered studies of potential diagnostic biomarkers and efforts to identify pathophysiologic subgroups. The greatest number of studies focused on spectral analysis as a potential diagnostic index with four out of 21 reporting a directionally similar finding as interpreted by Gurau et al. (2017). They concluded that, despite inconsistencies, some generalizations could be inferred. Significant differences in the alpha band were shown by five studies with relaxed eyes open condition. Four studies showed a decrease in absolute alpha spectral power in ASD in children of similar ages, but another showed elevated absolute alpha power in adults. Inspection of the cited studies reveal that even a common finding of “decrease in absolute spectral power” is unclear because absolute spectral power was not presented in each paper.

Specifically, selecting the only two studies among the four with supposedly common findings that included an ASD group of more than 25 subjects, one reports lower relative (not absolute) alpha power calculated from channels T3 + T6 + C3 + F4 (selected by stepwise discriminant-function analysis) (Chan et al., 2007) whereas another study used retrospective clinical recordings from a 10 year period (2001–2011) to look for differences in recordings from subjects diagnosed as ASD (children 4–8 years old). The control group was based on selecting EEGs that had been read as normal in same age children over the same period who based on chart review were free of any NDD although the reasons for EEGs having been done were not specified. This later study reported a lower ratio of posterior to anterior alpha power (Matlis et al., 2015). Relative advantages of examining absolute and relative power in a particular frequency bandwidth require empirical study.

At the current exploratory stage of the development of EEG biomarkers, investigators appear to be operating with the dual aim of discovery neuroscience and a secondary goal of finding or generating data suggestive that there might be something worth following as a biomarker. But from the vantage point of looking for biomarkers that might be informative at an individual level, for predicting something of clinical importance about a specific person, small, site-specific studies with varying analytic approaches and specific outcome measures make it difficult to select parameters to pursue for biomarker development with confidence. Developing such confidence will require multisite studies (using coordinated recruitment or at least some set of overlapping data) using common (standardized) measures that allow not only for apples to apples comparisons of results across sites but also, ideally, allowing for aggregation of data into common databases.

As an example of a systems neuroscience-based sensory ERP study, a recent study utilized an EEG measure that can applied across a genetic mouse model of a disorder and in patients with the disorder utilizing an ingenious aural chirp stimulus. To evaluate the ability of the brain to generate robust oscillatory responses across a 1–100 Hz frequency range, they evaluated neural synchronization across this range to an auditory stimulus oscillating from low to high frequency in the 1–100 Hz frequency range band. Deficits were detectable in the gamma frequency range in FXS patients (Ethridge et al., 2017) as they were later observed in fMR1 KO mice (Lovelace et al., 2018). The human study was a single site study in 17 subjects with full mutation FXS individuals (age range 13–57 of whom 4 were female) and 17 age/gender matched controls. Obviously, issues of potential age and gender effects would ultimately need to be addressed as well as what is usually required to move from a single site study in a small number of individuals to a broader population in diverse settings. Such issues will be partially resolved in the ongoing multisite NeuroNEXT study of the Novartis mGluR5 negative allosteric modulator AFQ056.

The investigators used an analytic approach including PCA-weighted un-baseline-corrected epoched single-trial data to generate single-trial power (STP) metrics which revealed decreased gamma band phase-locking to the chirp stimulus in FXS individuals. Interestingly, there were elevations of baseline gamma power in FXS *vs.* control subjects before, during and after chirp presentation as in fMR1 KO mice. This raises the question of what additional information is provided by the STP measure of the ability to synchronize neural oscillations to the frequency of the auditory stimulus relative to information provided by increased baseline gamma power from a predictive biomarker perspective, given the observed correlation between elevated baseline gamma power values and the reduced entrainment of gamma band activity to the chirp stimulus.

To advance these measures as potential biomarkers, one might begin with examining whether the two measures (baseline resting-state gamma-power and gamma-band STP to chirp) met a criteria of 90% test-retest reliability on the same day and over longer periods. A second issue is the examination of distributional characteristics of these alterations, such as whether there is a subgroup of highly deviant outliers or bimodality with discrete subgroups. This is needed to get a sense of the distribution of values at the individual level, something not provided by group-level heat-maps that displayed log power at neural oscillation frequencies over time (ms). To move from discovery science to establishing the promise and utility of the measures as biomarkers for advancing drug discovery, future studies will need to establish clinical relevance and study larger groups to reasonably estimate parameter distributions and ROC curves for the different metrics examined. Optimal electrodes to use for this work would also need to be formally determined and validated to maximize the signal to noise ratio of data in a consistent way across laboratories for individual study participants.

The third example considers ERP response to a psychological stimulus in studies of ASD. A recent meta-analysis of 23 studies (374 participants) established the finding of delayed N170 response to face stimuli in individuals with ASD (Kang et al., 2018). The N170 is a negative-going change in the ERP waveform that peaks approximately 170ms after stimulus presentation. In healthy individuals, it is larger in amplitude and shorter in latency to faces in comparison with responses to inanimate objects. As such, it is presumed to reflect neural activity associated with early-stage face processing, and believed to reflect aspects of social cognition. Overall deficits in N170 ERP amplitudes were not seen, but amplitudes were reduced in adults and those with higher cognitive ability relative to matched typically developing controls. Only 3 of the studies involved at least 25 subjects per group and the review utilized effect sizes of group differences calculated from each study. The extent to which the specific latencies or amplitudes did or did not align across studies is not addressed in the review and difficult to extract from the actual papers given differences in the details of the paradigms employed.

Neural indices of face processing are of interest as candidate biomarkers for social processes in ASD, and build on an extensive psychological literature linking face perception to social process in typically developing (TD) individuals and in ASD (Bublatzky et al., 2017; Webb et al., 2017). While overall effects are promising at the group level, potential limitations of this line of work include: (1) some non-confirming reports in the literature, (2) uncertainty about how much this deficit relates to early-stage visual system disturbances *vs.* later perceptual analysis of faces, (3) uncertainty about whether or how the effect is related to affective response to faces *vs.* a disturbance in the perceptual ability to process face information, (4) uncertainty about whether an index of this nature will separate subtypes of patients for stratification purposes or provide a dimensional/objective measure of a core behavioral trait in ASD with which the EEG measure is correlated—and therefore the additional information provided by the EEG metric, (5) the neural and cognitive implications of a delayed N170 component that is not reduced in amplitude remain to be fully elucidated, and (6) psychometric properties (reliability and validity) of the latency and amplitude measures with regard to establishing potential cut-off points at the individual level continue to be developed. Several of these issues are being addressed by the ongoing Autism Biomarkers Consortium for Clinical Trials (ABC-CT) study, a US-based multisite effort to identify biomarkers to support intervention research in autism (McPartland, 2016, 2017).

The study of N170 in ASD is rooted in psychological models and behavioral observations, and has the advantages of a relatively strong supporting literature and face-valid clinical relevance. The approach also has potential limitations, including limited potential for translational integration and limited clarity of neurobiological implications beyond localization of effect to particular areas of neocortex to be informative at a level that could provide direct rational link to drug targets. The reduced N170 latency in ASD is of interest in its own right, but the comparison to our auditory chirp example highlights the relative development, strengths and limitations of psychologically rooted and neurobiologically rooted approaches for developing EEG biomarkers for advancing drug discovery. Clarifying and maximally utilizing the relative advantages of these approaches for developing EEG biomarkers for neurodevelopmental disorders remains an important and relatively uncharted direction for future research.

What might represent a sufficient degree of standardization and what level of “assay” performance would one be looking for to rule a measure in or out as a usual biomarker for some specified COU? For any functional brain measure such as EEG, with many potential variables both as regards acquisition paradigms including number of channels and analytic approaches, the suitability of a range of approaches for different COU will require extensive evaluation. One would expect that approaches could be compared in later stage developmental efforts prior to large-scale validation. That does not mean, however, that it is not possible to specify some common practices that will allow the field to be more confident that the raw data generated at different sites does or does not replicate (same values, not simply directionally similar case-control differences). Multivariable development studies can ideally contrast distributional properties and differential utility in a COU of different EEG measures, and examine their relation to age and developmental state of the brain. This would allow for addressing questions of whether a biomarker can be informative at the individual level, which is crucial for their applied use. For that purpose, we suggest preliminary thresholds for promising biomarkers:

1. Deployable in >80% of patients administered by technician level staff outside of a CNS research center
2. Reproducible value of a specific EEG measurements within individual at ±7.5%, if tested within the same acquisition period
3. Day to day stability within an individual at ±10% in absence of change in clinical condition, treatment and environmental factors; stability week to week within ±15%
4. Evidence that different sites can achieve values in the same individual within 15% of each other (traveling subject approach) and generate mean value for a control group of 12 subjects within 10%
5. Normative data that allows for correction of data for any significant effects of age, gender, educational status or intellectual capacity that might influence measurement of an EEG biomarker

While reliability thresholds mathematically depend on effect sizes, many of these specific proposed degrees of variation of a variable within an individual reflect the experience of one of the authors (WZP) in terms of assumptions that go into powering of studies to assess the utility of potential biomarkers carried out within the Biomarkers Consortium of the Foundation of the National Institute of Health. The precise performance targets are illustrative and might be relaxed or tightened depending on the situation; the point is to have pre-specified and reasonably stringent performance targets when moving from biomarker discovery to qualification for some COU. If an EEG biomarker can meet the proposed targets, it should be relatively straight-forward to determine utility in a COU with a sample size on the order of 100–200 participants.

Given the complexity of the brain, and everything that contributes to EEGs, combinations of EEG measures may ultimately achieve the most stable and useful characterization of brain function within an individual. To identify the “best” parameter combinations, approaches such as machine learning, which benefit from larger sample sizes, can help identify biomarker measures that in combination optimize practical utility. In light of the above criteria for a single biomarker, criteria that a combination is “better” should involve at least a 5% increase in, for instance, the AUC of the ROC curve for some purpose of use.

We assume that to approach meeting these criteria, which are admittedly aspirational, standardization of paradigms, analysis pipelines, electrode array size and perhaps even equipment will be required. Recently completed and ongoing studies with EEG and ERP in various neurodevelopmental and psychiatric populations as well as in healthy volunteers as measures of drug effect have generated data that will help assess whether these criteria are met under ideal research conditions. If not, the data may allow for more informed setting of criteria or argue that we search for EEG/ERP paradigms that could meet those as proposed.

We assume that later stage validation studies would necessarily be multi-site. Single site studies would be focused on biomarker discovery such as some novel ERP paradigm or resting state EEG measure. In keeping with recommendations from the FDA (Amur et al., 2015), we believe that to address criteria 1–5 at any level is best done through collaborative consortia approaches with extensive data sharing. Full transparency allows for confidence in the data and expedites the rate of uptake of any biomarker that may facilitate the development of desperately needed treatments.

Given all the considerations discussed above, it seems fair to say that biomarker development for NDDs is not nearing the end stage of well validated and regular application, but we do now see the end of the beginning phase as we move from pure discovery to planning for testing validation for application. Many small-sample studies have found promising leads, especially for EEG/ERP biomarkers both for ASD and for related single gene disorders such as FXS. Future studies will also need to examine community populations not rigorously selected for mechanistic studies in academic medical centers but recruited to characterize a disorder as it exists in the population. Secondly, biomarkers will need to be evaluated in terms of their proximity to clinical symptoms *vs.* to biological disease mechanisms. Both are important, but most approaches will have greater relevant utility for one or the other purpose. For example, one might consider ERP studies of psychological features such as emotional face viewing as a promising diagnostic biomarker for ASD, as it is likely to be common across ASD cases given its close association with social cognition, which is a defining feature of the disorder. Alternatively, a study of theta-gamma coupling at rest, a more fundamental feature of brain physiology, might be more likely to resolve syndromal heterogeneity and be linked to the selective action of particular drugs in particular individuals.

This distinction has important implications for biomarker evaluation. A biomarker useful for identifying a meaningful subgroup in a population almost by necessity would fail as a diagnostic biomarker by virtue of its low sensitivity for the condition, and a biomarker with high sensitivity likely would have limited utility for identifying subgroups within a clinical syndrome. This idea is related to the idea of degeneracy as one moves from gene to molecular biology to local circuit networks to large-scale functional networks to behavior. Biomarkers at different places along this path are likely to serve different purposes and will need to be developed and evaluated in this context. For this reason, and others, different ERP paradigms and analysis approaches to the data may be suitable for different diagnostic and predictive purposes, and need to be evaluated within the limits of their intended COU.

At a practical level, electrophysiological biomarkers will need to be evaluated for utility across the age-span, across sexes and disorders, in relation to treatment outcome to different classes of medication, and across different hardware and software analysis strategies. Given the very large amount of data provided by resting-state and task-based analyses, novel analytic and signal-processing approaches recently developed to work with the data may allow for much more information content at the individual level not possible with currently employed data analytic pipelines. Addressing such issues in scale is now a major challenge for electrophysiological biomarker development in NDDs but one that holds enormous promise. By committing to standardization of some core set of measures, the field should be able to generate a new set of EEG/ERP derived measures that will better serve various COUs for developing treatments of NDDs.

## Author Contributions

All authors contributed intellectually to the concepts within the manuscript, framing, writing, and editing.

## Conflict of Interest Statement

The authors declare that the research was conducted in the absence of any commercial or financial relationships that could be construed as a potential conflict of interest.
